# Safety and Efficacy of Salvage Treatment for Recurrent Nasopharyngeal Carcinoma: A Single‐Center Retrospective Study Over 10 Years

**DOI:** 10.1002/cam4.71604

**Published:** 2026-02-15

**Authors:** Yanrong Luo, Boning Cai, Bo Li, Lei Du, Lin Ma

**Affiliations:** ^1^ Department of Radiation Oncology First Medical Center of Chinese PLA General Hospital Beijing China; ^2^ Department of Radiation Oncology Hainan Hospital of Chinese PLA General Hospital Sanya China; ^3^ Department of Cardiology Hainan Hospital of Chinese PLA General Hospital Sanya China

**Keywords:** locoregional, nasopharyngeal carcinoma, recurrence, re‐irradiation, salvage treatment

## Abstract

**Background:**

To analyze survival outcomes and identify prognostic factors in patients with locoregionally recurrent nasopharyngeal carcinoma (NPC) receiving salvage treatment, and to evaluate the safety profile of re‐irradiation.

**Methods:**

We retrospectively analyzed clinical data from 95 patients with recurrent NPC (rM0) who were diagnosed and received salvage treatment at the PLA General Hospital between January 2008 and October 2018. Patients were stratified into two treatment groups: the radiotherapy (RT) group (*n* = 72) and the non‐RT group (*n* = 23).

**Results:**

With a median follow‐up of 37 months (4–100 months), the 3‐year overall survival (OS), progression‐free survival (PFS), and disease‐specific survival (DSS) rates were 58.7%, 46.2%, and 33.3%, respectively. The corresponding 5‐year OS, PFS, and DSS rates were 34.2%, 31.3%, and 11.1%, respectively. Significant differences in survival outcomes were observed between the RT and non‐RT groups: OS (43.4% vs. 0%, *p* < 0.001), PFS (36.2% vs. 0%, *p* < 0.001), and DSS (16.2% vs. 0%, *p* = 0.007). Of the tumor‐related deaths, 19 (31.1%) were attributed to massive hemorrhage and 18 (29.5%) to distant metastasis. The univariate and multivariate analyses identified re‐irradiation as an independent predictor of superior OS and PFS, and salvage surgery as a predictor of OS improvement. Conversely, advanced nodal disease (rN2‐3) independently predicted worse OS and PFS, while local and regional recurrence associated with poorer PFS.

**Conclusion:**

Salvage re‐irradiation significantly improves survival outcomes in locoregionally recurrent NPC, whereas advanced nodal disease (rN2‐3) independently predicts poor prognosis. Massive hemorrhage and distant metastasis are the most common causes of death.

**Trial Registration:**

Clinical Trial Register: ChiCTR2500098189

## 1 Introduction

Nasopharyngeal carcinoma (NPC) is a unique head and neck malignancy with varied racial and geographic distribution, which is endemic in North Africa and Southeast Asia, most notably in South China [1]. Contemporary meta‐analyses integrating data from our institutional experience and multinational cohorts indicate that 10%–20% of NPC patients develop locoregional recurrence following primary treatment [1, 2, 3, 4, 5, 6, 7]. The treatment of recurrent NPC presents formidable clinical challenges due to anatomical constraints and cumulative radiation exposure [8, 9]. Therapeutic strategies must be individualized based on multidimensional parameters, including recurrence location, temporal interval from initial treatment, tumor invasion patterns, and patient performance status. Current salvage modalities encompass surgical resection, re‐irradiation, systemic chemotherapy, targeted therapies, and immunotherapy. While salvage surgery may achieve durable control in select cases with focal recurrence, its application is precluded in lesions involving critical neurovascular structures [10, 11]. As a highly radiosensitive malignancy, radiotherapy (RT) is the cornerstone of the treatment of primary NPC. For recurrent NPC, re‐irradiation through intensity‐modulated radiation therapy (IMRT), stereotactic body radiation therapy (SBRT), or interstitial brachytherapy is a key treatment modality. However, these approaches carry substantial risks of severe complications, including osteoradionecrosis, temporal lobe necrosis, and catastrophic hemorrhage, necessitating meticulous patient selection [12, 13]. Although recurrent NPC maintains chemosensitivity, long‐term survival outcomes with first‐line gemcitabine‐cisplatin regimens remain suboptimal [14, 15, 16]. Emerging evidence from phase II/III trials supports the therapeutic potential of immune checkpoint inhibitors, either as monotherapy or in combination with chemotherapy, demonstrating improved survival outcomes with favorable toxicity profiles in recurrent/metastatic settings [16, 17, 18, 19, 20]. To optimize salvage treatment strategies for locoregionally recurrent NPC, we conducted a retrospective cohort study to assess long‐term survival outcomes and identify prognostic factors in patients receiving salvage treatment, and to evaluate the safety profile of re‐irradiation.

## 2 Methods

### 2.1 Patient and Clinical Characteristics

We retrospectively reviewed the medical records, imaging data, and histopathological reports of patients who were diagnosed with locoregionally recurrent NPC after primary radiotherapy at the Department of Radiation Oncology, Chinese PLA General Hospital. Between January 2008 and October 2018, 107 patients were identified with local and/or regional recurrence after primary radiotherapy, either as monotherapy or in combination with chemotherapy. After excluding 12 patients who did not receive salvage treatment, 95 patients were included in the final analysis cohort. This study population was stratified into two groups based on treatment modality: the radiotherapy (RT) group (*n* = 72), comprising patients who underwent re‐irradiation, and the no‐radiotherapy (non‐RT) group (*n* = 23), consisting of patients who did not receive re‐irradiation. This study was approved by the Ethics Committee of Chinese PLA General Hospital (S2024‐531‐01).

Clinical data encompassing demographic characteristics (gender, age), medical history, presenting symptoms, radiographic findings, histopathological results, recurrence interval, recurrent tumor stage, salvage treatment modalities, treatment‐related complications, and outcomes at the last follow‐up were systematically collected. The diagnosis of recurrence was confirmed through magnetic resonance imaging (MRI) or computed tomography (CT) scans, supplemented by histopathological verification via endoscopic biopsy or ultrasound‐guided cervical lymph node biopsy when clinically indicated. A subset of patients underwent additional evaluation using positron emission tomography/computed tomography (PET/CT) for comprehensive staging assessment. Tumor staging was performed according to the standardized criteria outlined in the 2017 American Joint Committee on Cancer (AJCC) staging manual, 8th edition.

### 2.2 Re‐Irradiation

Re‐irradiation techniques employed in this study encompassed step‐and‐shoot IMRT, sliding window IMRT, Helical Tomotherapy, and SBRT. Treatment modality selection was individualized based on recurrent tumor characteristics (location and extent) and patient preferences. During the initial study period, step‐and‐shoot IMRT served as the primary re‐irradiation technique. Following the installation of a Tomotherapy accelerator at Chinese PLA General Hospital in 2009, Helical Tomotherapy became the predominant treatment modality for locoregionally recurrent NPC.

Patients were positioned supine with head and neck immobilized using a thermoplastic mask. Contrast‐enhanced CT images with 3 mm slice thickness were acquired for treatment planning and subsequently transferred to the Pinnacle3 8.0 workstation for image fusion. Target volume delineation was guided by contrast‐enhanced CT, MRI, or PET/CT imaging. The gross tumor volume (GTV) was defined as follows: GTVnx for locally recurrent tumors and GTVnd for regional recurrent lymph nodes. The planning target volume for primary tumor (pGTVnx) was generated by expanding GTVnx with a 3–5 mm margin, constrained by anatomical boundaries including the brainstem, spinal cord, optic chiasm, and optic nerves. For nodal disease, pGTVnd was created by applying a 3 mm margin to GTVnd. Prescription doses ranged 60–70 Gy to GTVnx and 66–70 Gy to GTVnd, delivered in 30–33 fractions. Two patients underwent stereotactic body radiotherapy (SBRT): one with intracranial and sphenoid sinus recurrence received 40 Gy in 8 fractions, while another with cavernous sinus recurrence was treated with 32 Gy in 8 fractions. One patient with right cervical lymph node recurrence received interstitial brachytherapy using radioactive seeds, without undergoing surgical resection of the affected lymph node. Re‐irradiation was strictly confined to recurrent tumor volumes and involved lymph nodes, without elective nodal irradiation.

### 2.3 Chemotherapy, Biological Targeted Therapy and Immunotherapy

Among the 95 patients analyzed, 83 patients (87.4%) underwent chemotherapy, 23 patients (24.2%) received biological targeted therapy, and 4 patients (4.2%) administered immunotherapy following disease recurrence. In the RT group, salvage chemotherapy was administered to 63 patients (87.5%), with the following treatment modalities: neoadjuvant chemotherapy (NACT) was administered to 46 patients (63.9%), concurrent chemotherapy (CCT) to 55 patients (76.4%), and adjuvant chemotherapy (AC) to only 7 patients (9.7%). The major regimens in NACT included TP (docetaxel 75 mg/m^2^ IV on Day 1 and cisplatin 80 mg/m^2^ IV on Day 1) or TPF (docetaxel 60 mg/m^2^ IV on Day 1, cisplatin 60 mg/m^2^ IV on Day 1 and 5‐fluorouracil 600 mg/m^2^ by continuous intravenous IV delivery over 120 h), administered for 2–3 cycles. The most commonly used platinum‐based CCT regimens consisted of single‐agent cisplatin (80–100 mg/m^2^ IV on Day 1) or TP (docetaxel 60–80 mg/m^2^ IV on Day 1 and cisplatin 60–80 mg/m^2^ IV on Days 1–3), administered for 1–3 cycles. Chemotherapy doses and cycles were slightly adjusted based on individual patient's adverse reactions. The treatment regimen was individualized based on patient characteristics, disease staging, and treatment tolerance, with the principle of no more than six cycles of total chemotherapy. Among the patients, 23 received biological targeted anti‐EGFR monoclonal antibody treatment: 17 patients (73.9%) were administered nimotuzumab 200 mg, five patients (21.7%) received cetuximab with a loading dose of 400 mg/m^2^ followed by 250 mg/m^2^ weekly, and one patient (4.4%) was treated with daily olaparib during re‐irradiation. Additionally, four patients received pembrolizumab (200 mg on Day 1, every three weeks for three cycles) during re‐irradiation.

### 2.4 Follow‐Up and Statistical Considerations

The patients underwent regular follow‐up evaluations to monitor their disease status, which included comprehensive assessments such as physical examinations, nasopharyngoscopy, chest CT, neck and abdominal ultrasonography, MRI and/or CT of the nasopharyngeal, and bone scans. The follow‐up schedule was structured as follows: every 3 months during the first 2 years post‐salvage treatment, every 6 months from Years 3 to 5, and annually thereafter. Key outcomes measured included progression‐free survival (PFS), DSS, and overall survival (OS). The duration was calculated from the date of salvage treatment start to the date of each event or last follow‐up. DSS was defined from the start of salvage treatment to the death caused by NPC or treatment‐related complications. Late toxicities were defined and graded according to the Radiation Therapy Oncology Group (RTOG) radiation morbidity scoring criteria and the Common Terminology Criteria for Adverse Events (Version 3.0) [21]. Statistical analyses were conducted to compare categorical variables using the *χ* ^*2*^ test and Fisher's exact test, while continuous variables were analyzed using the Mann–Whitney *U* test. Survival curves were generated, and multivariate survival analysis was performed using Kaplan–Meier and Cox regression analyses. The log‐rank test was employed to assess the significance of differences between groups. All statistical analyses were executed using SPSS software, version 26.0 (IBM Corp, Armonk, NY), and a *p*‐value < 0.05 was considered statistically significant.

## 3 Results

### 3.1 Patient Characteristics

Ninety‐five patients with local and/or regional recurrent NPC were included in the analysis. There were 67 males (70.5%) and 28 females (29.5%), with ages ranging from 24 to 81 years (median, 52 years). The interval from the completion of primary radiation therapy to the diagnosis of recurrence ranged from 5 to 338 months (median, 23 months). Among the patients, 49 patients (51.6%) were diagnosed with local recurrence, 31 patients (32.6%) with regional recurrence, and 15 patients (15.8%) with both local and regional recurrence. Of the 64 patients with local recurrence, nasopharyngeal recurrence was the most common subtype, observed in 49 patients (76.6%), followed by skull base recurrence in 31 patients (48.4%). For lymph node recurrence, 20 patients (43.5%) had involvement in the left neck, 18 patients (39.1%) in the right neck, and the remaining 8 patients (17.4%) exhibited bilateral involvement. Level‐II lymph nodes (76.1%) were the most frequently affected site, followed by the parotid region (23.9%). Based on the 2017 AJCC staging system, 55 patients (57.9%) were classified as rT0‐2, while 40 patients (42.1%) were classified as rT3‐4. Additionally, 85 patients (89.5%) were staged as rN0‐1, and 10 patients (10.5%) as rN2‐3. Patients were further divided into two groups based on their acceptance of re‐irradiation: 72 patients were assigned to the RT group, and 23 patients to the non‐RT group. The median time to recurrence was 32 months (range: 6–338 months) in the RT group and 18 months (range: 5–51 months) in the non‐RT group, with a statistically significant difference was observed in the time to recurrence between the two groups (*p* = 0.010). Surgical interventions were performed in one patient in the RT group (nasal endoscopic resection) and three patients in the non‐RT group (surgical resection), with a significant difference between the two groups (*p* = 0.012). No significant differences were observed in baseline characteristics, including age, gender, primary radiotherapy, primary chemotherapy, recurrent type, recurrent T stage, recurrent N stage, or salvage chemotherapy. The clinical characteristics and salvage treatments of the study patients are summarized in Table 1. All patients underwent salvage treatment after recurrence. Re‐irradiation techniques, doses, and other salvage treatments for patients in the RT group are summarized in Table 2.

**TABLE 1:** Clinical characteristics and salvage treatments among the study patients.

Characteristics	RT group (*n* = 72)		Non‐RT group (*n* = 23)		*p*
	No. %		No. %		
Age (years)					
< 50	30	41.7	10	43.5	0.878
≥ 50	42	58.3	13	56.5	
Gender					
Female	22	30.6	6	26.1	0.682
Male	50	69.4	17	73.9	
Primary radiotherapy					
2D/3D‐CRT	13	18.1	2	8.7	0.457
IMRT	59	81.9	21	91.3	
Primary radiotherapy dose (Gy)					
< 66	6	8.3	1	4.3	> 0.99
≥ 66	66	91.7	22	95.7	
Primary chemotherapy cycle					
< 4	46	63.9	14	60.9	0.794
≥ 4	26	36.1	9	39.1	
Recurrent type					
Local	36	50.0	13	56.5	0.616
Regional	23	31.9	8	34.8	
Locoregional	13	18.1	2	8.7	
Recurrent T stage					
rT0‐2	41	56.9	14	60.9	0.740
rT3‐4	31	43.1	9	39.1	
Recurrent N stage					
rN0‐1	64	88.9	21	91.3	> 0.99
rN2‐3	8	11.1	2	8.7	
Time to recurrence (years)					
< 2	31	58.3	17	86.9	0.010
≥ 2	41	41.7	6	13.1	
Salvage chemotherapy					
No	9	12.5	3	13.0	> 0.99
Yes	63	87.5	20	87.0	
Salvage chemotherapy cycle					
< 4	34	47.2	7	30.4	0.157
≥ 4	38	52.8	16	69.6	
Salvage surgery					
No	71	98.6	19	82.6	0.012
Yes	1	1.4	4	17.4	

Abbreviations: 2D/3D‐CRT, Two/three‐dimensional conformal radiotherapy; IMRT, intensity‐modulated radiotherapy; rN stage, recurrent N stage; rT stage, recurrent T stage.

**TABLE 2:** Re‐irradiation techniques, doses, and other salvage treatments for patients in the RT group.

Treatment	No. (*n* = 72)	%
Re‐irradiation technique		
IMRT	69	95.8
SBRT	2	2.8
Brachytherapy	1	1.4
Re‐irradiation technique for recurrent type		
Local recurrence	36	
IMRT	34	94.4
SBRT	2	5.6
Regional recurrence	23	
IMRT	22	95.7
Brachytherapy	1	4.3
Locoregional recurrence	13	
IMRT	13	100.0
Re‐irradiation dose		
Brachytherapy	1	1.4
SBRT 30–40 Gy in 8 fractions	2	2.8
IMRT 60–66 Gy	7	9.7
IMRT 67.5 Gy	1	1.4
IMRT 69–70 Gy	61	84.7
Other salvage treatment		
C	37	51.4
C + B	21	29.1
C + I	3	4.2
C + B + I	1	1.4
S + C + B	1	1.4
N	9	12.5

Abbreviations: B, biological targeted therapy; C, chemotherapy; I, immunotherapy; IMRT, intensity‐modulated radiotherapy; N, none treatment; S, surgery; SBRT, stereotactic body radiotherapy.

### 3.2 Survival Outcomes

As of the last follow‐up date on 31 March 31 2020, the median follow‐up duration was 37 months (range: 4–100 months). Four patients were lost to follow‐up at 6, 10, 15, and 48 months after re‐irradiation. Among the 61 deceased patients (RT group: 42; non‐RT group: 19), 54 (88.5%) died from tumor‐related causes, while 7 (11.5%) died of other diseases or accidents. The most common cause of death among all deceased patients was massive hemorrhage following salvage treatment, occurring in 19 cases (overall: 19/61, 31.1%; RT group: 10/42, 23.8%; non‐RT group: 9/19, 47.3%). In the RT group, all 10 patients who received re‐irradiation (IMRT: 9; SBRT: 1) developed massive hemorrhages within the re‐irradiation fields (skull base, nasopharynx or neck) without evidence of tumor progression or recurrence after salvage re‐irradiation. These hemorrhages were attributed to radiation‐induced vascular injury (carotid blowout). All 9 patients in the non‐RT group (including 2 who underwent salvage surgery) experienced massive hemorrhages at sites of recurrent disease during or after salvage therapy, confirming tumor progression as the causative factor. Other causes of death included distant metastasis in 18 patients (29.5%), local or regional failure in 11 patients (18.0%), and treatment‐related in six patients (9.8%). A statistically significant difference was observed between the RT and non‐RT groups in terms of tumor‐related deaths (*p* = 0.032). Specifically, distant metastasis was the leading cause of death in the RT group, whereas massive hemorrhage was the predominant cause in the non‐RT group. No significant difference was noted in deaths attributed to other diseases or accidents after salvage treatment (*p* = 0.292). The causes of death for the 61 patients are summarized in Table 3.

**TABLE 3:** Causes of death among the 61 patients.

Cause of death	Radiotherapy		No‐radiotherapy		*p*
	(*n* = 42)		(*n* = 19)		
	No. %		No. %		
Tumor‐related^a^ (*n* = 54)					
Local or regional failure	6	14.3	5	26.3	0.258
Distant metastasis	17	40.5	1	5.3	0.006
Massive hemorrhage	10	23.8	9	47.3	0.066
Treatment‐related	4	9.5	2	10.5	> 0.99
Total	37	88.1	17	89.4	0.711
Other diseases or accidents (*n* = 7)					
Other malignant tumors	2	4.8	0	0.0	> 0.99
Pneumonia	2	4.8	1	5.3	> 0.99
Cardiocerebrovascular events	1	2.4	1	5.3	> 0.99
Total	5	11.9	2	10.5	> 0.99

^a^ Local or regional failure: death attributed to local or regional tumor progression (excluding massive hemorrhage) after salvage therapy; Distant metastasis: death from metastatic disease after salvage therapy; Massive hemorrhage: death due to massive hemorrhage secondary to either radiation‐induced necrosis or local or regional tumor progression; Treatment‐related: death caused by salvage therapy‐induced systemic organ failure.

**FIGURE 1:**
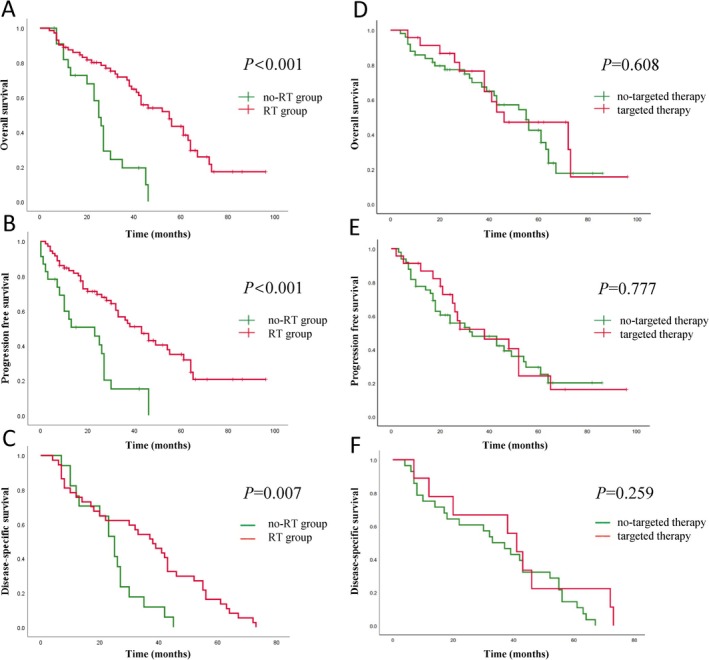
Kaplan–Meier survival curves were generated to compare outcomes between the RT group and the non‐RT group (A–C), as well as between patients with and without targeted therapy (D–F). Specifically: (A, D) Overall survival (OS); (B, E) Progression‐free survival (PFS); (C, F) Disease‐specific survival (DSS).

Among all 95 patients, the 3‐year PFS, OS, and DSS rates were 46.2%, 58.7%, and 33.3%, respectively, while the 5‐year rates were 31.3%, 34.2%, and 11.1%. In the RT group (72 patients), the 3‐year PFS, OS, and DSS rates were 54.5%, 71.8%, and 54.1%, respectively, and the 5‐year rates were 36.2%, 43.4%, and 16.2%. In contrast, in the non‐RT group (23 patients), the 3‐year PFS, OS, and DSS rates were 15.2%, 19.4%, and 11.8%, respectively. Notably, none of the patients in the non‐RT group survived beyond 5 years. Statistically significant differences were observed in OS between the two groups. The 5‐year actuarial OS rates were 43.4% for the RT group and 0% for the non‐RT group (log‐rank *p* = 0.000; Figure 1A), while the 3‐year rates were 71.8% and 19.4%, respectively. The median survival was 40.5 months in the RT group and 25 months in the non‐RT group. Similarly, significant differences were found in PFS outcomes between the RT and non‐RT groups (log‐rank *p* = 0.000; Figure 1B). The median PFS was 29 months in the RT group and 18 months in the non‐RT group. The 3‐year and 5‐year PFS rates were 54.5% and 36.2% in the RT group, compared to 15.2% and 0% in the non‐RT group. For DSS, the RT group also demonstrated significantly higher rates than the non‐RT group (log‐rank *p* = 0.007; Figure 1C). The median DSS was 38 months in the RT group and 25 months in the non‐RT group. The 3‐year DSS rates were 54.1% and 11.8% for the RT and non‐RT groups, respectively, while the 5‐year DSS rates were 16.2% and 0%.

Twenty‐three patients received targeted therapy during salvage treatment, all of whom were in the RT group. The 5‐year OS rates for patients with targeted therapy and those without were 47.0% and 42.4%, respectively, showing no statistically significant difference (log‐rank *p* = 0.608; Figure 1D). Similarly, the 5‐year PFS rates for patients with targeted therapy and those without were 24.3% and 29.4%, respectively, with no statistically significant difference (log‐rank *p* = 0.777; Figure 1E). For DSS, the 5‐year DSS rates for patients with targeted therapy and those without were 22.2% and 14.3%, respectively, again showing no statistically significant difference (log‐rank *p* = 0.259; Figure 1F).

### 3.3 Univariate and Multivariate Analysis of Prognostic Factors

OS, PFS, and DSS were modeled using regression analysis with potential prognostic factors in both univariate and multivariate models. We analyzed age, gender, recurrent pattern, recurrent T stage, recurrent N stage, time to recurrence, re‐irradiation, and salvage surgery as prognostic factors for survival in all patients. In univariate analysis, re‐irradiation significantly correlated with improved OS, PFS, and DSS (all *p* < 0.001), while salvage surgery showed association with better OS (*p* = 0.006) and DSS (*p* < 0.001). Variables demonstrating *p* < 0.15 were included in multivariate analysis, which confirmed re‐irradiation as an independent predictor for superior OS, PFS, and DSS, while salvage surgery for OS and DSS improvement. Conversely, rN2‐3 disease independently predicted worse OS, PFS, and DSS, while local and regional recurrence was associated with poorer PFS. Prognosis factors significantly associated in the multivariate analysis are shown in Table 4.

**TABLE 4:** Prognosis factors significantly associated with outcomes in the multivariate analysis.

Prognosis factors	OS		PFS		DSS	*p*
	HR (95% CI)	*p*	HR (95% CI)	*p*	HR (95% CI)	
Re‐irradiation, (yes vs. no)	0.252 (0.135–0.474)	< 0.001	0.322 (0.172–0.601)	< 0.001	0.124 (0.050–0.308)	< 0.001
Salvage surgery, (yes vs. no)	0.220 (0.067–0.725)	0.013	0.332 (0.102–1.080)	0.067	0.165 (0.049–0.556)	0.004
Recurrent type, (local and regional vs. local or regional)	2.260 (0.876–5.835)	0.092	2.853 (1.114–7.312)	0.029	2.645 (0.824–8.490)	0.102
rN stage, (rN0‐1 vs. rN2‐3)	0.280 (0.118–0.664)	0.004	0.306 (0.129–0.727)	0.007	0.135 (0.047–0.386)	< 0.001
Time to recurrence (year), (< 2 vs. ≥ 2)	1.064 (0.628–1.802)	0.818	1.156 (0.672–1.992)	0.600	0.913 (0.440–1.894)	0.807

Abbreviations: CI, confidence interval; DSS, disease‐specific survival; HR, hazard ratio; OS, overall survival; PFS, progression‐free survival; rN stage, recurrent N stage.

### 3.4 Failure and Late Radiation‐Related Complications

Local or regional failure was defined as either persistent disease (uncontrolled) or recurrence after salvage treatment. Fourteen patients (14.7%) experienced local or regional failure, with a median time to the occurrence of 6.5 months (range: 0–55 months). Among these cases, 11 patients had died, including seven with uncontrolled disease and four with recurrent disease. Local or regional failure primarily occurred in the non‐RT group, with nine patients who received salvage chemotherapy alone experiencing local or regional uncontrolled disease.

Furthermore, distant metastasis was observed in 21 patients (22.1%). Lung and bone metastasis accounted for the majority of these cases, with 12 patients (57.1%) developing lung metastasis and 10 patients (47.6%) developing bone metastasis. A total of 18 patients died, including 17 patients in the RT group and only one patient in the non‐RT group. The median time to metastasis occurrence was 21 months (range: 4–65 months).

Based on the patient's symptoms and imaging findings during follow‐up, the most common late radiation‐related complications were xerostomia, subcutaneous fibrosis, hearing impairment, and visual impairment. The incidence rates of these late complications are summarized in Table 5.

**TABLE 5:** Late complications observed in 72 patients after re‐irradiation.

Late complications	No.	%
Xerostomia	61	84.7
Subcutaneous fibrosis	48	66.7
Hearing impairment	33	45.8
Visual impairment	21	29.2
Dysphagia	19	26.4
Massive hemorrhage	10	23.8
Trismus	6	8.3
Radioactive caries	5	6.9
Radiation encephalopathy	2	2.8

## 4 Discussion

A significant proportion of patients with recurrent NPC can achieve long‐term survival through aggressive salvage treatment, primarily involving re‐irradiation and surgical resection. The survival rate of patients with locally recurrent NPC who undergo aggressive treatment is significantly higher than that of patients receiving chemotherapy alone or symptomatic support [22]. Surgical salvage is most suitable for early‐stage disease. However, if the recurrent tumor in the nasopharynx extends beyond the pharyngobasilar fascia (T2b and above), complete tumor resection without damaging adjacent major vessels becomes challenging [12]. Currently, the majority of patients opt for re‐irradiation, including IMRT, SBRT, and brachytherapy. Re‐irradiation has therapeutic benefits for recurrent NPC, with reported 5‐year OS rates ranging from 30% to 50% [2, 22, 23]. In this study, we observed that the 5‐year OS, PFS, and DSS rates in the RT group were 43.4%, 36.2%, and 16.2%, respectively, while no patients in the non‐RT group survived beyond 5 years. The 5‐year OS, PFS, and DSS rates in the RT group were significantly superior to those in the non‐RT group (OS: *p* < 0.001; PFS: *p* < 0.001; DSS: *p* = 0.007). Univariate analysis revealed that patients who underwent re‐irradiation after recurrence exhibited significantly improved survival outcomes compared to those who did not receive re‐irradiation, suggesting that salvage re‐irradiation plays a crucial role in enhancing post‐recurrence survival. These findings align with existing empirical evidence [22], further supporting the role of re‐irradiation in improving survival outcomes for patients with recurrent NPC.

Successful re‐irradiation for recurrent NPC relies on precise tumor delineation and accurate dose delivery to the gross recurrent tumor volume. Conventional RT is often challenging due to the proximity of critical structures that have already received high doses during initial treatment. Owing to its dosimetric advantages over 2D/3D conformal radiotherapy (CRT), IMRT is now widely used and recommended as the primary re‐irradiation modality [24]. In our study, 72 out of 95 patients with recurrent NPC (75.8%) underwent re‐irradiation. Among them, 69 patients (95.8%) received IMRT, two patients (rT4N0) received SBRT, and one patient (rT0N1) received brachytherapy, confirming IMRT as the most commonly used re‐irradiation technique. Wu et al. [25] reported that fractionated stereotactic radiotherapy (FSRT) for patients with locally recurrent NPC achieved a complete response (CR) rate of 64%, a partial response (PR) rate of 26%, and an overall response rate of 90%. SBRT is an effective salvage treatment for local failure in NPC, often administered alone or as a boost following external beam RT [25, 26]. Another study reported a 5‐year local control rate (LCR) of up to 85% after brachytherapy for recurrent NPC [27]. Therefore, for small and localized recurrent tumors, both SBRT and brachytherapy are viable options.

Previous studies recommend a dose of at least 60 Gy (in equivalent dose at 2 Gy fractions EQD2) for the GTV as the optimal dose for the second course of radiotherapy, aiming to balance tumor control and risk of complications [24]. IMRT enables delivery of doses exceeding 60 Gy to recurrent tumors, achieving promising 5‐year local control rate (LCR) up to 80.7% [28]. However, doses below 60 Gy reduce the 5‐year LCR to 52.0% [29]. Conversely, doses ≥ 68 Gy have been associated with decreased post‐re‐irradiation survival due to severe toxicities [23, 30]. Higher re‐irradiation doses may increase fatal complications without improving survival outcomes, as the risks of toxicity may outweigh the benefits of tumor control. Therefore, the recommended total dose for re‐irradiation using IMRT is 60–66 Gy [24]. In this study, 69 patients with recurrent NPC in the RT group underwent IMRT, with the radiation dose to the recurrent GTVs exceeding 60 Gy in all cases. Of these patients, 61 (88.4%) received higher dose levels of 69–70 Gy. Notably, the majority of patients in this study were administered re‐irradiation doses that exceeded the conventional recommended range of 60–66 Gy, which may be associated with the observed higher incidence and severity of radiation‐related adverse effects. These findings underscore the importance for radiation oncologists to meticulously optimize total radiation dose when developing re‐irradiation protocols for recurrent NPC, with the goal of achieving an optimal balance between therapeutic efficacy and safety.

Newly diagnosed NPC usually has cervical lymph node metastasis, predominantly involving level II (97.9%) and level‐III (46.0%), with over 50% of cases showing multi‐level involvement (≥ 2 levels) [31]. In regional recurrence cases, nodal distribution patterns differ significantly, with level II remaining the most frequent site (67.7%), followed by level‐III (22.8%), while less than one‐third exhibit multi‐level involvement [32]. In our current study, we observed similar patterns of lymph nodal recurrence, with level‐II (76.1%, 35/46) being the most frequent site of recurrence, followed by the parotid region (23.9%, 11/46). Only 34.8% (16/46) of recurrent cases showed multi‐level involvement. These findings align with previous reports that recurrent NPC demonstrates less extensive nodal distribution than primary tumors. Liu et al. [32] demonstrated no significant survival differences between selective neck dissection (SND) and comprehensive neck dissection (CND) in regional recurrence cases. Current evidence supports SND as the preferred salvage re‐irradiation for residual neck disease due to its comparable efficacy to CND and excellent regional control in selected patients [2]. Accordingly, our treatment strategy employed targeted re‐irradiation of recurrent nodes without prophylactic neck irradiation.

Re‐irradiation has been shown to improve the survival prognosis of patients with recurrent NPC. However, the incidence and severity of radiation‐related toxicities must be carefully considered. These toxicities include skull base osteonecrosis, arterial rupture leading to massive bleeding, and mucosal necrosis. Such complications not only significantly impair patients' quality of life but also constitute a major cause of mortality. Lee et al. [2] identified carotid blowout as a significant cause of treatment‐related deaths, alongside mucosal and adjacent soft tissue/bone necrosis that leads to profuse bleeding. Leong et al. [23] reported that the incidence of grade 3 or higher hemorrhage (from the internal carotid artery or its branches as a result of mucosal necrosis) reached 19% following re‐irradiation. In our analysis of mortality causes among 61 patients in this study, massive hemorrhage and distant metastasis emerged as the predominant causes of death, with incidences of 31.1% (19/61) and 29.5% (18/61), respectively. The incidence of hemorrhage‐related mortality following re‐irradiation in our study was higher than that reported by Leong et al. [23], but not higher than that in the non‐RT group (23.8% vs. 47.3%, *p* = 0.066). Therefore, focal re‐irradiation confined to recurrent tumors and/or metastatic lymph nodes does not significantly increase the risk of fatal hemorrhage.

In this study, only five patients underwent salvage surgery before 2011. Among them, three patients underwent surgery alone (two with rT4N0 and one with rT0N1), one patient (rT3N0) received concurrent chemoradiotherapy post‐surgery, and one patient (rT0N1) underwent chemotherapy following surgery. After salvage treatment, three patients (two who underwent surgery alone and one who received surgery plus chemotherapy) died from massive local bleeding. Additionally, one patient experienced brainstem recurrence and died of systemic failure after surgery combined with concurrent chemoradiotherapy, while one patient was lost to follow‐up 6 months after surgery. The median time of death for four patients was 12 months (range: 6–30 months). Univariate and multivariate analyses revealed that salvage surgery after recurrence is associated with OS. However, due to the limited number of patients undergoing salvage surgery (only five patients, accounting for 5.3% of the cohort), the median survival time post‐surgery was only 1 year. This limited sample size does not provide sufficient evidence to establish OS benefits from salvage surgery.

After excluding contraindications of chemotherapy, salvage chemotherapy remains the most common treatment for recurrent NPC. Platinum‐based chemotherapy is currently the first‐line therapy for recurrent NPC [15, 33]. However, the outcome of patients with advanced disease remains poor, as they often develop resistance to standard chemotherapy over time. In the early 21st century, targeted therapy and immunotherapy were not conventional salvage treatments for recurrent NPC. In this study, 24.2% of patients received targeted therapy, while only 4.2% underwent immunotherapy. Within the RT group, no statistically significant differences were observed in 5‐year OS and PFS between patients who received targeted therapy and those who did not (OS: 47.0% vs. 42.4%, *p* = 0.608; PFS: 24.3% vs. 29.4%, *p* = 0.777). Currently, targeted therapy and immunotherapy, particularly the latter, play a crucial role in the comprehensive management of recurrent NPC, especially immunotherapy. Compared to the traditional gemcitabine plus cisplatin regimen [15, 33], the addition of immune checkpoint inhibitors has demonstrated significantly prolonged PFS in the first‐line treatment for recurrent or metastatic NPC [16, 17, 18, 19]. Immunotherapy has not only improved survival outcomes for patients with recurrent and metastatic NPC but also offers a manageable safety profile [20, 34, 35, 36]. Currently, the combination of toripalimab (a PD‐1 inhibitor) with gemcitabine and cisplatin has become a new standard first‐line regimen for recurrent or metastatic NPC. Maintenance therapy with S‐1 or capecitabine following first‐line chemotherapy significantly enhanced OS and PFS in patients with recurrent or metastatic NPC [37].

Consistent with prior evidence linking advanced rN stage to adverse outcomes [32, 38], our multivariate analysis not only reaffirmed its independent prognostic value in recurrent NPC but also highlighted the pronounced survival detriment associated with rN2‐3 disease (OS/PFS, both *p* < 0.01). These findings underscore the need for risk stratification based on nodal status in recurrent settings.

While this retrospective analysis provides valuable real‐world evidence regarding salvage treatment outcomes, several limitations should be acknowledged. First, as a single‐center study from a low‐prevalence region, our findings may be subject to potential patient selection bias. The proportion of patients with early recurrence was significantly higher in the non‐RT group, likely because radiation oncologists tend to avoid re‐irradiation after a recent prior RT course due to safety concerns. As early recurrence typically reflects more aggressive tumor biology and poorer prognosis, this imbalance may have confounded survival comparisons between groups. Furthermore, potential confounding variables (including performance status, comorbidities, and recurrent tumor characteristics) were not systematically balanced between cohorts, a known limitation of retrospective studies. These factors may collectively compromise the comparability of survival outcomes between the RT and non‐RT groups. Second, the assessment of late complications was predominantly based on patients' subjective reports during follow‐up visits, since standardized scoring criteria were not consistently documented in medical records for all patients. Consequently, the severity grading of late complications could not be systematically assessed. Third, although propensity score matching or inverse probability weighting would have better controlled for baseline imbalances between treatment groups than multivariate Cox regression, their implementation was prevented by sample size constraints and incomplete treatment records for externally referred patients. These limitations underscore the need for further validation through multicenter studies or prospective randomized controlled trials to enhance the robustness and generalizability of our conclusions. We propose a future prospective trial comparing combined‐modality therapy (incorporating local interventions [surgery or standardized IMRT/VMAT re‐irradiation at 60–66 Gy EQD_2_] with systemic therapy) versus systemic therapy alone. This study would employ rigorous stratification by performance status, comorbidities, and tumor characteristics (volume, location, and recurrence pattern) to enable comprehensive evaluation of both therapeutic efficacy and toxicity profiles.

In conclusion, our study showed that salvage re‐irradiation significantly improves survival outcomes in locoregionally recurrent NPC, whereas advanced nodal disease (rN2‐3) independently predicts poor prognosis. Massive hemorrhage and distant metastasis are the most common causes of death.

## Author Contributions

**Yanrong Luo:** writing – original draft, investigation, data curation, visualization, validation, formal analysis. **Boning Cai:** data curation, investigation, formal analysis, supervision. **Bo Li:** writing – original draft, data curation, validation, formal analysis, visualization, investigation. **Lei Du:** data curation, investigation, writing – review and editing, supervision. **Lin Ma:** conceptualization, methodology, supervision, project administration, writing – review and editing, data curation, investigation, validation, formal analysis, visualization.

## Funding

The authors have nothing to report.

## Ethics Statement

All procedures followed the ethical standards of the institutional and/or national research committee and the 1964 Declaration of Helsinki, including its later amendments or equivalent ethical standards. This study was approved by the Ethics Committee of Chinese PLA General Hospital (S2024‐531‐01). The study was approved by the principal investigators and study group.

## Consent

Written consent was not required for this type of study, in accordance with Chinese's Ethical Guidelines for Medical and Biological Research Involving Human Subjects. Registry and the Registration No. of the study/trial: Chinese.

## Conflicts of Interest

The authors declare no conflicts of interest.

## Data Availability

The data that support the findings of this study are available from the corresponding author upon reasonable request.

## References

[1] K. C. W. Wong , E. P. Hui , K. W. Lo , et al., “Nasopharyngeal Carcinoma: An Evolving Paradigm,” Nature Reviews. Clinical Oncology 18, no. 11 (2021): 679–695, 10.1038/s41571-021-00524-x. 34194007

[2] A. W. M. Lee , W. T. Ng , J. Y. W. Chan , et al., “Management of Locally Recurrent Nasopharyngeal Carcinoma,” Cancer Treatment Reviews 79 (2019): 101890, 10.1016/j.ctrv.2019.101890. 31470314

[3] K. H. Au , R. K. C. Ngan , A. W. Y. Ng , et al., “Treatment Outcomes of Nasopharyngeal Carcinoma in Modern Era After Intensity Modulated Radiotherapy (IMRT) in Hong Kong: A Report of 3328 Patients (HKNPCSG 1301 Study),” Oral Oncology 77 (2018): 16–21, 10.1016/j.oraloncology.2017.12.004. 29362121

[4] L. Du , X. X. Zhang , L. Ma , et al., “Clinical Study of Nasopharyngeal Carcinoma Treated by Helical Tomotherapy in China: 5‐Year Outcomes,” BioMed Research International 2014 (2014): 980767, 10.1155/2014/980767. 25114932 PMC4119915

[5] Y. P. Liu , Y. H. Wen , J. Tang , et al., “Endoscopic Surgery Compared With Intensity‐Modulated Radiotherapy in Resectable Locally Recurrent Nasopharyngeal Carcinoma: A Multicentre, Open‐Label, Randomised, Controlled, Phase 3 Trial,” Lancet Oncology 22, no. 3 (2021): 381–390, 10.1016/S1470-2045(20)30673-2. 33600761

[6] Z. Peng , Y. Wang , R. Fan , et al., “Treatment of Recurrent Nasopharyngeal Carcinoma: A Sequential Challenge,” Cancers (Basel) 14, no. 17 (2022): 4111, 10.3390/cancers14174111. 36077648 PMC9454547

[7] Y. H. Chen , S. D. Luo , S. C. Wu , et al., “Clinical Characteristics and Predictive Outcomes of Recurrent Nasopharyngeal Carcinoma‐A Lingering Pitfall of the Long Latency,” Cancers (Basel) 14, no. 15 (2022): 3795, 10.3390/cancers14153795. 35954458 PMC9367553

[8] J. S. Li , P. Blanchard , C. H. L. Wong , et al., “International Recommendations on Postoperative Management for Potentially Resectable Locally Recurrent Nasopharyngeal Carcinoma,” International Journal of Radiation Oncology, Biology, Physics 120 (2024): 1294–1306, 10.1016/j.ijrobp.2024.07.2143. 39009321

[9] J. J. Juarez‐Vignon Whaley , M. Afkhami , M. Onyshchenko , et al., “Recurrent/Metastatic Nasopharyngeal Carcinoma Treatment From Present to Future: Where Are we and Where Are we Heading?,” Current Treatment Options in Oncology 24, no. 9 (2023): 1138–1166, 10.1007/s11864-023-01101-3. 37318724 PMC10477128

[10] M. K. Chen , J. C. Lai , C. C. Chang , and M. T. Liu , “Minimally Invasive Endoscopic Nasopharyngectomy in the Treatment of Recurrent T1‐2a Nasopharyngeal Carcinoma,” Laryngoscope 117, no. 5 (2007): 894–896, 10.1097/MLG.0b013e3180381644. 17473691

[11] W. I. Wei , “Salvage Surgery for Recurrent Primary Nasopharyngeal Carcinoma,” Critical Reviews in Oncology/Hematology 33, no. 2 (2000): 91–98, 10.1016/s1040-8428(99)00069-4. 10737370

[12] Y. M. Tian , Y. Guan , W. W. Xiao , et al., “Long‐Term Survival and Late Complications in Intensity‐Modulated Radiotherapy of Locally Recurrent T1 to T2 Nasopharyngeal Carcinoma,” Head and Neck 38, no. 2 (2016): 225–231, 10.1002/hed.23880. 25244494

[13] E. Newton , D. Valenzuela , J. Foley , A. Thamboo , and E. Prisman , “Outcomes for the Treatment of Locoregional Recurrent Nasopharyngeal Cancer: Systematic Review and Pooled Analysis,” Head and Neck 43, no. 12 (2021): 3979–3995, 10.1002/hed.26836. 34403174

[14] L. Zhang , Y. Huang , S. Hong , et al., “Gemcitabine Plus Cisplatin Versus Fluorouracil Plus Cisplatin in Recurrent or Metastatic Nasopharyngeal Carcinoma: A Multicentre, Randomised, Open‐Label, Phase 3 Trial,” Lancet 388, no. 10054 (2016): 1883–1892, 10.1016/s0140-6736(16)31388-5. 27567279

[15] S. Hong , Y. Zhang , G. Yu , et al., “Gemcitabine Plus Cisplatin Versus Fluorouracil Plus Cisplatin as First‐Line Therapy for Recurrent or Metastatic Nasopharyngeal Carcinoma: Final Overall Survival Analysis of GEM20110714 Phase III Study,” Journal of Clinical Oncology 39, no. 29 (2021): 3273–3282, 10.1200/JCO.21.00396. 34379443 PMC8500603

[16] Y. Yang , J. Pan , H. Wang , et al., “Tislelizumab Plus Chemotherapy as First‐Line Treatment for Recurrent or Metastatic Nasopharyngeal Cancer: A Multicenter Phase 3 Trial (RATIONALE‐309),” Cancer Cell 41, no. 6 (2023): 1061–1072, 10.1016/j.ccell.2023.04.014. 37207654

[17] H. Q. Mai , Q. Y. Chen , D. Chen , et al., “Toripalimab or Placebo Plus Chemotherapy as First‐Line Treatment in Advanced Nasopharyngeal Carcinoma: A Multicenter Randomized Phase 3 Trial,” Nature Medicine 27, no. 9 (2021): 1536–1543, 10.1038/s41591-021-01444-0. 34341578

[18] H. Q. Mai , Q. Y. Chen , D. Chen , et al., “Toripalimab Plus Chemotherapy for Recurrent or Metastatic Nasopharyngeal Carcinoma: The JUPITER‐02 Randomized Clinical Trial,” Journal of the American Medical Association 330, no. 20 (2023): 1961–1970, 10.1001/jama.2023.20181. 38015220 PMC10685882

[19] Y. Yang , S. Qu , J. Li , et al., “Camrelizumab Versus Placebo in Combination With Gemcitabine and Cisplatin as First‐Line Treatment for Recurrent or Metastatic Nasopharyngeal Carcinoma (CAPTAIN‐1st): A Multicentre, Randomised, Double‐Blind, Phase 3 Trial,” Lancet Oncology 22, no. 8 (2021): 1162–1174, 10.1016/S1470-2045(21)00302-8. 34174189

[20] F. H. Wang , X. L. Wei , J. Feng , et al., “Efficacy, Safety, and Correlative Biomarkers of Toripalimab in Previously Treated Recurrent or Metastatic Nasopharyngeal Carcinoma: A Phase II Clinical Trial (POLARIS‐02),” Journal of Clinical Oncology 39, no. 7 (2021): 704–712, 10.1200/jco.20.02712. 33492986 PMC8078488

[21] J. D. Cox , J. Stetz , and T. F. Pajak , “Toxicity Criteria of the Radiation Therapy Oncology Group (RTOG) and the European Organization for Research and Treatment of Cancer (EORTC),” International Journal of Radiation Oncology, Biology, Physics 31, no. 5 (1995): 1341–1346, 10.1016/0360-3016(95)00060-C. 7713792

[22] Y. P. Chen , A. T. C. Chan , Q. T. Le , P. Blanchard , Y. Sun , and J. Ma , “Nasopharyngeal Carcinoma,” Lancet 394, no. 10192 (2019): 64–80, 10.1016/S0140-6736(19)30956-0. 31178151

[23] Y. H. Leong , Y. Y. Soon , K. M. Lee , L. C. Wong , I. W. K. Tham , and F. C. H. Ho , “Long‐Term Outcomes After Reirradiation in Nasopharyngeal Carcinoma With Intensity‐Modulated Radiotherapy: A Meta‐Analysis,” Head and Neck 40, no. 3 (2018): 622–631, 10.1002/hed.24993. 29130584

[24] W. T. Ng , Y. L. Soong , Y. C. Ahn , et al., “International Recommendations on Reirradiation by Intensity Modulated Radiation Therapy for Locally Recurrent Nasopharyngeal Carcinoma,” International Journal of Radiation Oncology, Biology, Physics 110, no. 3 (2021): 682–695, 10.1016/j.ijrobp.2021.01.041. 33571626

[25] S. X. Wu , D. T. Chua , M. L. Deng , et al., “Outcome of Fractionated Stereotactic Radiotherapy for 90 Patients With Locally Persistent and Recurrent Nasopharyngeal Carcinoma,” International Journal of Radiation Oncology, Biology, Physics 69, no. 3 (2007): 761–769, 10.1016/j.ijrobp.2007.03.037. 17601682

[26] G. Ozyigit , M. Cengiz , G. Yazici , et al., “A Retrospective Comparison of Robotic Stereotactic Body Radiotherapy and Three‐Dimensional Conformal Radiotherapy for the Reirradiation of Locally Recurrent Nasopharyngeal Carcinoma,” International Journal of Radiation Oncology, Biology, Physics 81, no. 4 (2011): e263–e268, 10.1016/j.ijrobp.2011.02.054. 21514737

[27] S. C. Law , W. K. Lam , M. F. Ng , S. K. Au , W. T. Mak , and W. H. Lau , “Reirradiation of Nasopharyngeal Carcinoma With Intracavitary Mold Brachytherapy: An Effective Means of Local Salvage,” International Journal of Radiation Oncology, Biology, Physics 54, no. 4 (2002): 1095–1113, 10.1016/s0360-3016(02)03009-2. 12419437

[28] Y. J. Hua , F. Han , L. X. Lu , et al., “Long‐Term Treatment Outcome of Recurrent Nasopharyngeal Carcinoma Treated With Salvage Intensity Modulated Radiotherapy,” European Journal of Cancer 48, no. 18 (2012): 3422–3428, 10.1016/j.ejca.2012.06.016. 22835782

[29] L. Koutcher , N. Lee , M. Zelefsky , et al., “Reirradiation of Locally Recurrent Nasopharynx Cancer With External Beam Radiotherapy With or Without Brachytherapy,” International Journal of Radiation Oncology, Biology, Physics 76, no. 1 (2010): 130–137, 10.1016/j.ijrobp.2009.01.055. 19467802

[30] Y. Q. Li , Y. M. Tian , S. H. Tan , et al., “Prognostic Model for Stratification of Radioresistant Nasopharynx Carcinoma to Curative Salvage Radiotherapy,” Journal of Clinical Oncology 36, no. 9 (2018): 891–899, 10.1200/jco.2017.75.5165. 29412781

[31] Y. Sun , J. Ma , T. X. Lu , et al., “Regulation for Distribution of Metastatic Cervical Lymph Nodes of 512 Cases of Nasopharyngeal Carcinoma,” Ai Zheng 23, no. 11 Suppl (2004): 1523–1527. 15566672

[32] Y. P. Liu , H. Li , R. You , et al., “Surgery for Isolated Regional Failure in Nasopharyngeal Carcinoma After Radiation: Selective or Comprehensive Neck Dissection,” Laryngoscope 129, no. 2 (2019): 387–395, 10.1002/lary.27317. 30325027

[33] P. Bossi , A. T. Chan , C. Even , J. P. Machiels , and ESMO Guidelines Committee , “ESMO‐EURACAN Clinical Practice Guideline Update for Nasopharyngeal Carcinoma: Adjuvant Therapy and First‐Line Treatment of Recurrent/Metastatic Disease,” Annals of Oncology 34, no. 3 (2023): 247–250, 10.1016/j.annonc.2022.11.011. 36529446

[34] Y. Yang , T. Zhou , X. Chen , et al., “Efficacy, Safety, and Biomarker Analysis of Camrelizumab in Previously Treated Recurrent or Metastatic Nasopharyngeal Carcinoma (CAPTAIN Study),” Journal for Immunotherapy of Cancer 9, no. 12 (2021): 3790, 10.1136/jitc-2021-003790. PMC8693086 34933967

[35] H. Huang , Y. Yao , X. Deng , et al., “Immunotherapy for Nasopharyngeal Carcinoma: Current Status and Prospects (Review),” International Journal of Oncology 63, no. 2 (2023): 97, 10.3892/ijo.2023.5545. 37417358 PMC10367053

[36] S. Wang , X. Huang , R. Li , Z. Zhou , and M. Kang , “Immune Checkpoint Inhibitor Combined With Chemotherapy Versus Chemotherapy Alone in the First‐Line Treatment for Recurrent or Metastatic Nasopharyngeal Carcinoma: A Meta‐Analysis of Random Controlled Trials,” European Archives of Oto‐Rhino‐Laryngology 281 (2024): 5111–5118, 10.1007/s00405-024-08768-w. 38914820

[37] L. Hu , Y. Huang , and J. Zhang , “Maintenance Treatment With Oral Anticancer Agents After First‐Line Chemotherapy in Patients With Recurrent or Metastatic Nasopharyngeal Carcinoma: A Systematic Review and Meta‐Analysis,” European Archives of Oto‐Rhino‐Laryngology 282, no. 2 (2025): 589–595, 10.1007/s00405-024-08920-6. 39198304

[38] F. Han , C. Zhao , S. M. Huang , et al., “Long‐Term Outcomes and Prognostic Factors of Re‐Irradiation for Locally Recurrent Nasopharyngeal Carcinoma Using Intensity‐Modulated Radiotherapy,” Clinical Oncology (Royal College of Radiologists) 24, no. 8 (2012): 569–576, 10.1016/j.clon.2011.11.010. 22209574

